# *Pediococcus acidilactici* PA53 Supplementation Improves Stool Form and Modulates Gut Microbiota and Inflammatory Responses in Adults With Functional Constipation: A Randomized, Double‐Blind, Placebo‐Controlled Trial

**DOI:** 10.1002/fsn3.72370

**Published:** 2026-10-01

**Authors:** Yuhan Xia, Yongmei Cai, Yongwei Zhang, Rui Cai, Hanshu Liu, Zhiwen Qiang

**Affiliations:** ^1^ Clinical Nutrition Department General Hospital of Ningxia Medical University Yinchuan Ningxia China; ^2^ Laboratory Medical Center, General Hospital of Ningxia Medical University Yinchuan Ningxia China; ^3^ Class of 2021, Clinical Medicine, Third Clinical College Ningxia Medical University Yinchuan Ningxia China; ^4^ Class of 2023, Clinical Medicine, First Clinical College Ningxia Medical University Yinchuan Ningxia China

**Keywords:** constipation, gut microbiota, inflammation, *Pediococcus acidilactici* PA53, probiotic

## Abstract

This study aimed to evaluate the efficacy and safety of *Pediococcus acidilactici* ( *P. acidilactici* ) PA53 in adults with functional constipation. In this randomized, double‐blind, placebo‐controlled trial, 87 adults with functional constipation were assigned to receive either *P. acidilactici* PA53 (3.0 × 10^10^ CFU/day) or a dextrin placebo in a 1:1 ratio for 8 weeks. The primary outcome was improvement in constipation symptoms, assessed by the Bristol Stool Form Scale (BSFS). Secondary outcomes included spontaneous bowel movements (SBMs), quality of life, serum biomarkers of intestinal function, inflammatory markers, and gut microbiota composition via 16S rRNA sequencing. At week 8, the PA53 group showed a significantly higher BSFS score than placebo (2.76 ± 0.93 vs. 2.03 ± 0.85, *p* = 0.004) and a significant within‐group improvement from baseline (*p* = 0.001), indicating stool softening. The proportion of participants achieving 4–6 SBMs/week increased significantly in the PA53 group (from 16.7% to 38.1%, *p* = 0.028), but the between‐group difference was not significant (*p* = 0.144). Within the PA53 group, motilin and interleukin‐10 increased, while somatostatin and interleukin‐6 decreased (all *p* < 0.05); however, no between‐group differences were significant for any biomarker. Gut microbiota analysis revealed marked enrichment of *Pediococcus* and changes in microbial diversity in the PA53 group. PA53 was well tolerated with no adverse events. Overall, *P. acidilactici* PA53 safely improved stool form and was associated with beneficial changes in gut microbiota and inflammatory markers in adults with functional constipation. Effects on bowel movement frequency were not significant between groups and warrant further investigation.

**Clinical Trial Registration:** NCT06761443 (ClinicalTrials.gov)

## Graphical abstract

In this randomized, double‐blind, placebo‐controlled trial, 8‐week supplementation with *Pediococcus acidilactici* PA53 significantly improved stool form in adults with functional constipation. PA53 supplementation was associated with changes in inflammatory markers and gut microbiota, including increased Pediococcus abundance.

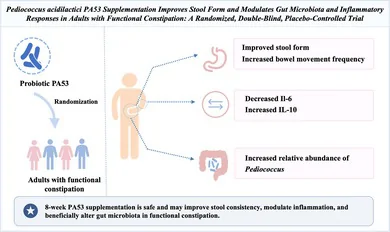

## 1 Introduction

Functional constipation (FC) is a common chronic bowel disorder typically characterized by infrequent bowel movements (less than three per week), hard stools (Bristol Stool Form Scale Types 1–2), and a feeling of incomplete evacuation, which significantly affects quality of life (Wang et al. 2023). The global prevalence of FC in adults is estimated to be around 10%–14% (Barberio et al. 2021). In older populations, prevalence can be as high as 30% (Fragakis et al. 2018; Salari et al. 2023). Even according to the Rome IV diagnostic criteria, FC remains a heterogeneous condition with marked differences in etiology and response to treatment (Palsson et al. 2016).

The pathophysiology of FC is multifactorial and includes impaired colonic motility, visceral hypersensitivity, dysregulation of the gut–brain axis and psychological factors (Vriesman et al. 2020; Erhardt et al. 2023). In recent years, mechanisms related to the microbiota have attracted increasing attention. Dysbiosis in FC is characterized by reduced numbers of short‐chain fatty acid‐producing bacteria, increased numbers of potentially pro‐inflammatory taxa, and decreased microbial diversity, potentially impairing mucosal motility, water absorption, and immunity, and contributing to low‐grade systemic inflammation (Wang et al. 2025; Ge et al. 2026). Several studies have reported decreased abundance of *Bifidobacterium* and *Lactobacillus* in FC, while certain pathogenic taxa increased, suggesting that therapeutic strategies may benefit from targeted modulation of the gut microbiota (Zhang et al. 2021).

Conventional treatment of FC includes lifestyle changes (increasing fiber and water intake, regular exercise), laxatives (osmotic or stimulant), and in refractory cases, prokinetic or secretagogues such as plecanatide, linaclotide, or lubiprostone (Lacy et al. 2016; Bharucha and Lacy 2020). While these interventions may be effective in some patients, they are limited by poor long‐term adherence, variable efficacy, and potential side effects, prompting interest in microbiota‐targeted complementary strategies such as probiotics, prebiotics, and synbiotics (Dimidi et al. 2014; Markowiak and Śliżewska 2017).

Probiotics—defined as “live microorganisms that, when administered in sufficient quantity, confer a health benefit to the host”—have shown modest promise in the treatment of FC (Hill et al. 2014). Several randomized controlled trials and meta‐analyses suggest that certain strains such as *Bifidobacterium lactis* HN019 and *Weizmannia coagulans* BC99 can improve stool frequency, shorten colonic transit time, and soften stool consistency (Ala‐Jaakkola et al. 2025; Fan et al. 2025). However, the effects of probiotics remain inconsistent, with considerable heterogeneity among studies due to variations in probiotic strains, intervention protocols, and study populations. A meta‐analysis of randomized controlled trials suggested that probiotics may provide benefits for certain constipation‐related outcomes; however, the overall evidence remains limited and further high‐quality studies are warranted to clarify strain‐specific efficacy (van der Schoot et al. 2022).

*Pediococcus acidilactici* is a Gram‐positive, homofermentative lactic acid bacterium commonly associated with fermented foods and has been widely studied for its technological properties and potential probiotic characteristics. Previous studies have suggested that certain *P. acidilactici* strains may possess functional properties, including tolerance to gastrointestinal environmental conditions and immunomodulatory potential; however, evidence regarding its clinical efficacy in humans remains limited (Takata et al. 2011; Papagianni and Anastasiadou 2009). In addition, *P. acidilactici* produces the bacteriocin pediocin, which has antimicrobial activity and may contribute to the maintenance of microbial homeostasis (Qiao et al. 2024). Despite promising preclinical results, high‐quality randomized controlled human trials integrating clinical, inflammatory, and microbiome endpoints for *P. acidilactici* are still lacking.

In this study, we conducted an 8‐week, randomized, double‐blind, placebo‐controlled trial to investigate the efficacy and safety of daily supplementation with *P. acidilactici* PA53 in adults with FC. The study also investigated potential mechanisms related to inflammatory markers and gut microbiota modulation. Primary outcomes included Bristol Stool Form Scale (BSFS) scores, while secondary outcomes included spontaneous bowel movements (SBMs), participant quality of life and psychological status, biochemical markers associated with bowel function, inflammatory markers, and composition of the gut microbiota. This study aims to close the mechanistic evidence gap for probiotics in FC and provide a basis for the potential use of *P. acidilactici* PA53 in clinical practice.

## 2 Materials and Methods

### 2.1 Study Design and Ethical Approval

This randomized, double‐blind, placebo‐controlled clinical trial was conducted at Ningxia Medical University General Hospital (Ningxia, China), from August 1, 2024, to May 30, 2025. The Ethics Committee of Ningxia Medical University General Hospital approved the study protocol (approval No. KYLL‐2024‐1715), and the study was conducted in accordance with the Declaration of Helsinki. All participants provided written informed consent prior to any study‐related procedures. The trial was registered at ClinicalTrials.gov (NCT06761443). The study followed the Consolidated Standards of Reporting Trials reporting guideline.

### 2.2 Participants

Functional constipation was defined according to the Rome IV diagnostic framework. Adults with diagnosed constipation were screened for eligibility. Inclusion criteria were: (1) age 18–70 years; (2) fewer than three SBMs per week and/or BSFS Type 1 or 2; (3) ability to comply with study requirements; (4) provision of signed informed consent; and (5) for participants of childbearing potential (including both sexes), agreement to use effective contraception from 14 days before screening until 6 months after study completion. For women, this included oral contraceptives, intrauterine devices, or barrier methods; for men, condom use or abstinence. Exclusion criteria were: (1) consumption of food or drugs known to affect gastrointestinal function (e.g., laxatives, probiotics) within 4 weeks prior to enrollment (Martoni et al. 2019); (2) a change in dietary habits within the past month or planned during the study; (3) pregnancy, lactation, or intention to become pregnant; (4) history of severe cardiovascular, pulmonary, hepatic, renal, metabolic (e.g., diabetes, thyroid disease), malignant, or immunological disorders; (5) antibiotic use within 4 weeks prior to the study (Martoni et al. 2019); (6) severe psychiatric illness; or (7) any other condition deemed by the investigators to make the participant unsuitable for the trial.

### 2.3 Sample Size Calculation

The sample size was calculated based on the primary outcome of change in stool consistency measured by the BSFS. Referring to a similar 12‐week probiotic trial in chronic constipation (Salo et al. 2024), which reported a between‐group difference of 0.74 points on the BSFS (intervention group change +0.51 vs. placebo group change −0.23) with a pooled standard deviation of 0.70, we assumed an effect size (Cohen's d) of approximately 1.06. Using a two‐sided significance level (α) of 0.05 and a statistical power (1−β) of 80%, the calculated sample size was 15 participants per group according to the standard formula for two independent means. To account for an anticipated dropout rate of 20% and to ensure sufficient power for secondary outcomes (e.g., gut microbiota and serum biomarkers), we aimed to enroll 40 participants per group (total 80). Ultimately, 87 participants were enrolled (44 in the probiotic group, 43 in the placebo group) to further enhance the robustness of the study.

### 2.4 Randomization, Blinding, and Compliance

Eighty‐seven eligible participants were randomly assigned in a 1:1 ratio to receive either *P. acidilactici* PA53 or an identical placebo. The randomization sequence was generated by an independent statistician using a computer‐based random number generator with a permuted block design (block size of 4), stratified by study center. Allocation concealment was achieved using sequentially numbered, opaque, sealed envelopes prepared by an individual not involved in the study; the envelopes were opened only after the participants had completed all baseline assessments. The study was double‐blinded: Participants, investigators, outcome assessors, and data analysts were all unaware of the group allocation until the database was locked. Compliance was assessed at each visit by counting the number of returned sachets and reviewing participant diaries that recorded daily intake. Participants were considered compliant if they consumed ≥ 80% of the prescribed sachets over the 8‐week period.

### 2.5 Intervention

Participants in the PA53 group received a daily dose of 3 g of dextrin blended with 3.0 × 10^10^ CFU of *P. acidilactici* PA53, whereas those in the placebo group received 3 g of dextrin alone without any probiotic. Both products were provided in identical sachets and were indistinguishable in terms of appearance, taste, and packaging. All participants were instructed to consume one sachet daily for 8 weeks, ideally at a consistent time each day. They were also asked to maintain their habitual diet and physical activity levels and to refrain from taking any other probiotics or medications known to influence bowel habits throughout the study period.

### 2.6 Outcome Measures

The primary outcome was improvement in stool form assessed by the BSFS. SBMs were recorded in participants' diaries and classified as an ordinal categorical variable (1–3, 4–6, and ≥ 7 bowel movements per week) and were included as a key secondary outcome. Secondary outcomes also included participants' quality of life and psychological status assessed by the Patient Assessment of Constipation Quality of Life (PAC‐QOL) questionnaire and the Depression, Anxiety and Stress Scale‐21 (DASS‐21); biochemical markers including serum 5‐hydroxytryptamine (5‐HT), motilin (MTL), and somatostatin (SS); inflammatory markers including interleukin 4 (IL‐4), interleukin 6 (IL‐6), and interleukin 10 (IL‐10); and gut microbiota composition analyzed by 16S rRNA gene sequencing (including alpha diversity, beta diversity, and differential abundance of taxa). Questionnaires were completed at baseline (Week 0) and Week 8, while biological samples were collected at baseline, week 4, and week 8.

### 2.7 Sample Collection and Storage

Approximately 5–10 g of fresh stool was collected in sterile tubes (Sarstedt, Germany) containing a nucleic acid stabilization solution at each fixed time point. The samples were stored at −20°C in the participants' homes and transported to the laboratory within 24 h in insulated containers with cold packs. Upon arrival, the samples were aliquoted into sterile cryovials under aseptic conditions and stored at −80°C until DNA extraction.

### 2.8 DNA Extraction and 16S rRNA Gene Sequencing

Genomic DNA was extracted from 200 mg of fecal material using the QIAamp Fast DNA Stool Mini Kit (Qiagen, Germany) according to the manufacturer's instructions. DNA quantity and purity were determined using a NanoDrop 2000 spectrophotometer (Thermo Fisher Scientific, USA). The hypervariable regions V3‐V4 of the bacterial 16S rRNA gene were amplified with primers 341F and 806R. PCR amplification (30 μL reactions) contained 15 μL of Phusion High‐Fidelity PCR Master Mix (New England Biolabs, USA), 0.2 μM of each primer and 10 ng of template DNA. Thermal cycles: initial denaturation at 98°C for 1 min; 30 cycles of denaturation at 98°C for 10 s, annealing at 50°C for 30 s, extension at 72°C for 30 s; final extension at 72°C for 5 min. Amplicons were run on 2% agarose gels, purified using the Qiagen Gel Extraction Kit and quantified using a Qubit 2.0 fluorometer. The sequencing libraries were prepared and sequenced on an Illumina MiSeq platform (Illumina, USA) using the paired‐end configuration (2 × 300 bp).

### 2.9 Bioinformatics and Statistical Analyses

Raw paired‐end reads were processed with USEARCH v11.0 (https://www.drive5.com/usearch/). After demultiplexing, reads were merged (minimum overlap 16 bp, maximum mismatch 0.1) and quality filtered (expected error rate ≤ 1.0, sequence length ≥ 400 bp). Chimeras were removed with UCHIME (Edgar 2013). High‐quality sequences were clustered into operational taxonomic units (OTUs) using the UPARSE algorithm at 97% similarity, and representative sequences were assigned to a taxonomy using the RDP classifier (confidence threshold 0.7) based on the SILVA 138 database. Alpha diversity indices (ACE, Chao1, Shannon) and beta diversity (Bray–Curtis distances) were calculated in R 4.2.0 with the packages vegan (Oksanen et al. 2022) and microeco (Liu et al. 2021). Differences in beta diversity were tested using permutative multivariate analysis of variance (PERMANOVA, function adonis in vegan) and visualized using principal coordinate analysis (PCoA). The relative abundances at phylum and genus levels were plotted, and differentially abundant taxa were identified using LEfSe analysis (Segata et al. 2011), which applies a nonparametric Kruskal–Wallis test (*p* < 0.05) followed by linear discriminant analysis (LDA score > 2.5) to estimate effect size.

Continuous variables were expressed as mean ± standard deviation (SD) and categorical variables as frequency (percentage). Comparisons between groups were performed using independent‐samples *t*‐tests or Mann–Whitney U‐tests for continuous variables, and χ^2^ or Fisher exact tests for categorical variables. Within‐group comparisons used paired *t*‐tests or Wilcoxon signed‐rank tests. The primary outcome (change in BSFS score) was tested at the two‐sided α = 0.05 level. SBM frequency and other secondary outcomes were analyzed accordingly. For secondary outcomes involving multiple comparisons (e.g., microbiota differential abundance), the Benjamini–Hochberg false discovery rate correction was applied. A *p*‐value < 0.05 was considered statistically significant unless otherwise specified. All statistical analyses were performed using R 4.2.0.

## 3 Results

### 3.1 Participant Demographics and Baseline Characteristics

Of the 87 participants randomized, 43 were assigned to the placebo group and 44 to the PA53 group (probiotic group). A total of 81 participants completed the 8‐week intervention: 39 in the placebo group and 42 in the PA53 group. In the placebo group, 2 participants were lost to follow‐up and 2 withdrew due to personal reasons. In the PA53 group, 2 participants withdrew due to personal reasons (Figure 1). At baseline, there were no statistically significant differences between the two groups in age, gender, BSFS score, SBMs, or blood parameters including serum albumin, alanine aminotransferase, and aspartate aminotransferase (all *p* > 0.05, Table 1).

**FIGURE 1:**
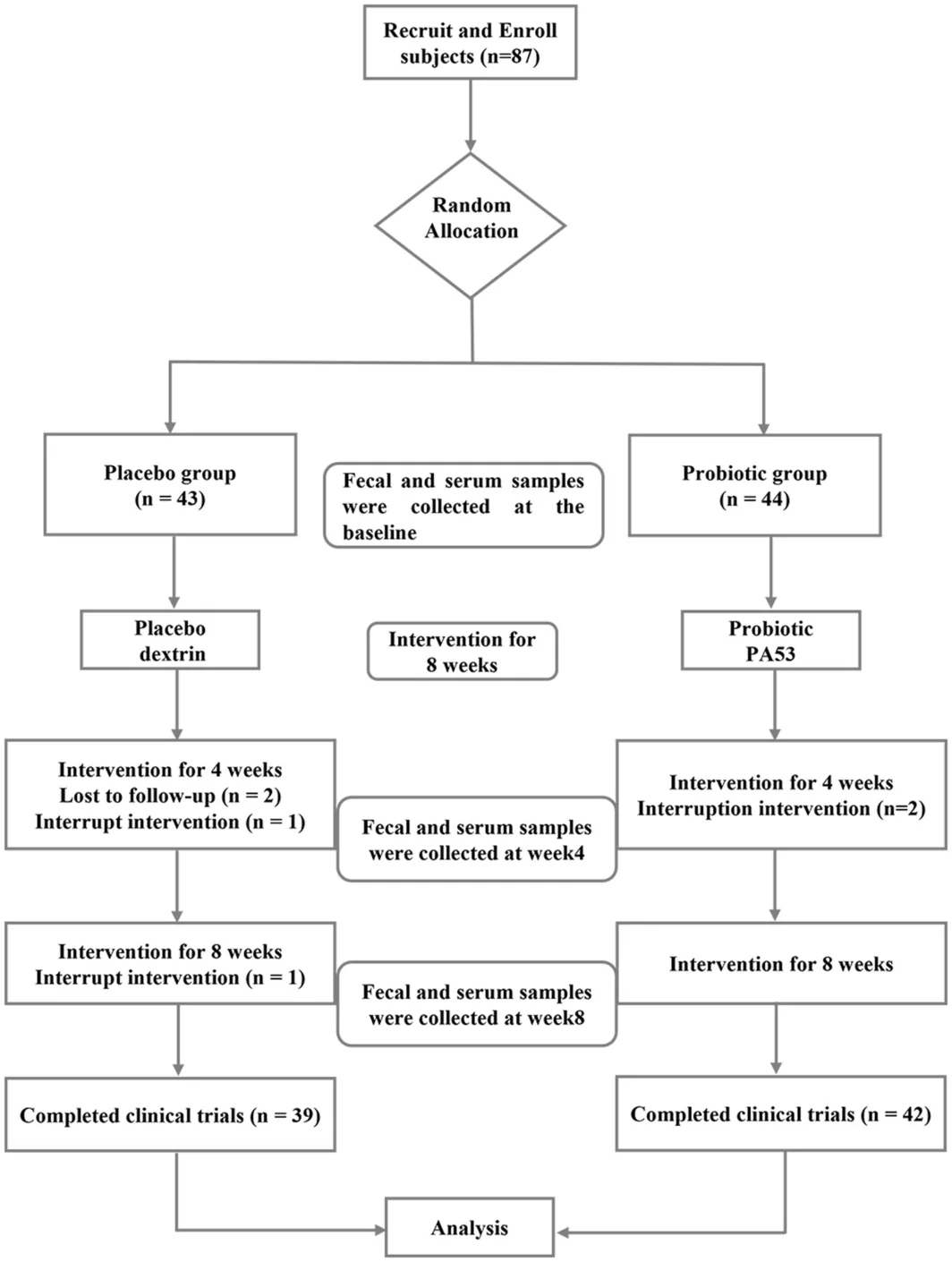
Clinical study flowchart.

**TABLE 1:** Baseline demographic and clinical characteristics of the study participants.

	Placebo group (*n* = 39)	PA53 group (*n* = 42)	*p*
Age	45.3 ± 12.1	42.4 ± 16.9	0.414
Gender			0.694
Male	7	9	
Female	32	33	
BSFS	2.00 ± 0.87	1.93 ± 0.80	0.753
SBM			0.626
1–3 times/week	34 (87.2%)	35 (83.3%)	
4–6 times/week	5 (12.8%)	7 (16.7%)	
Alb	45.41 ± 2.57	44.5 ± 2.55	0.114
ALT	20.74 ± 9.69	22.07 ± 9.24	0.334
AST	20.56 ± 5.73	21.67 ± 4.37	0.154
Urea	5.21 ± 1.66	5.22 ± 1.47	0.982
UA	275.92 ± 80.48	287.26 ± 72.8	0.359
Crea	64.65 ± 13.17	63.72 ± 11.96	0.741
GLU	4.54 ± 0.58	4.61 ± 0.74	0.441

*Note:* Values are presented as mean ± SD for continuous variables and *n* (%) for categorical variables. *p*‐values represent between‐group comparisons at baseline. *p*‐values were adjusted using the Benjamini–Hochberg false discovery rate (FDR) correction for multiple comparisons. Adjusted *p*‐values < 0.05 were considered statistically significant.

Abbreviations: Alb, Albumin; ALT, alanine aminotransferase; AST, aspartate aminotransferase; GLU, Glucose; UA, uric acid.

### 3.2 Changes in Stool Shape and Frequency

As shown in Table 2, after the 8‐week intervention, the PA53 group exhibited significant improvement in stool form, with BSFS scores markedly increased from baseline (from 1.93 ± 0.80 to 2.76 ± 0.93, *p* = 0.001), while no significant change was observed in the placebo group. At week 8, the BSFS score in the PA53 group was significantly higher than that in the placebo group (2.76 ± 0.93 vs. 2.03 ± 0.85, *p* = 0.004). Regarding bowel movement frequency, the PA53 group showed a within‐group improvement, with an increased proportion of participants reporting 4–6 bowel movements per week (from 7 at baseline to 16 at week 8, *p* = 0.028). However, no significant between‐group difference was observed in SBM distribution (*p* = 0.144). No significant changes were observed in the placebo group (*p* = 0.238). Furthermore, neither PAC‐QOL nor DASS‐21 scores showed significant changes in either group (*p* > 0.05, Table 2).

**TABLE 2:** Changes in stool characteristics, bowel frequency, quality of life and emotional state in the placebo and PA53 groups.

Variable	Placebo group		PA53 group		*p*			
	W0	W8	W0	W8	PA53_W0. vs. Placebo_W0	Placebo W0. vs. W8	PA53 W0. vs. W8	PA53_W8. vs. Placebo_W8
BSFS	2.00 ± 0.87	2.03 ± 0.85	1.93 ± 0.80	2.76 ± 0.93	0.753	0.438	0.001	0.004
SBMs					0.626	0.238	0.028	0.144
1–3 times/week	34 (87.18%)	30 (76.92%)	35 (83.33%)	26 (61.90%)				
4–6 times/week	5 (12.82%)	9 (23.08%)	7 (16.67%)	16 (38.10%)				
PAC‐QOL	39.10 ± 13.87	36.25 ± 14.71	40.21 ± 17.90	32.71 ± 14.52	0.811	0.440	0.139	0.780
DASS‐21	11.62 ± 7.72	13.90 ± 8.80	12.10 ± 10.43	11.00 ± 7.80	0.723	0.138	0.354	0.754

*Note:* Values are presented as mean ± SD for continuous variables and *n* (%) for categorical variables. *p* values represent within‐group comparisons between Week 0 and Week 8 in the placebo and PA53 groups, and between‐group comparisons at week 8 between the placebo and PA53 groups. *p*‐values were adjusted using the Benjamini–Hochberg false discovery rate (FDR) correction for multiple comparisons. Adjusted *p*‐values < 0.05 were considered statistically significant.

Abbreviations: BFFS, Bristol Stool Form Scale; DASS‐21, Depression Anxiety Stress Scale‐21; PAC‐QOL, Patient Assessment of Constipation Quality of Life questionnaire; SBMs, Spontaneous Bowel Movements.

### 3.3 Gut‐Related Biochemical and Immune Markers

We explored the effects of PA53 on gut function‐related biomarkers. For gut hormones, both groups showed similar trends over time, and no significant between‐group differences were observed for 5‐HT, MTL, or SS (all *p* > 0.05) (Figure 2). Between‐group comparisons were used as the primary basis for treatment effect evaluation. However, notable within‐group patterns emerged: MTL progressively increased in the PA53 group, with a 62.72% rise from baseline to week 8 (from 44.56 ± 41.35 pg/mL to 72.51 ± 51.43 pg/mL, *p* < 0.001), while the placebo group showed a 20.27% rise (from 54.03 ± 44.63 pg/mL to 64.98 ± 36.36 pg/mL, *p* < 0.001) (Figure 2B). Similarly, SS decreased by 33.94% in the PA53 group (from 20.95 ± 11.42 pg/mL to 13.84 ± 11.76 pg/mL, *p* < 0.001) compared with 20.26% in the placebo group (from 18.36 ± 3.84 pg/mL to 14.64 ± 11.41 pg/mL, *p* = 0.008) (Figure 2C). These within‐group changes reflect temporal patterns and should not be interpreted as evidence of differential treatment effects.

**FIGURE 2:**
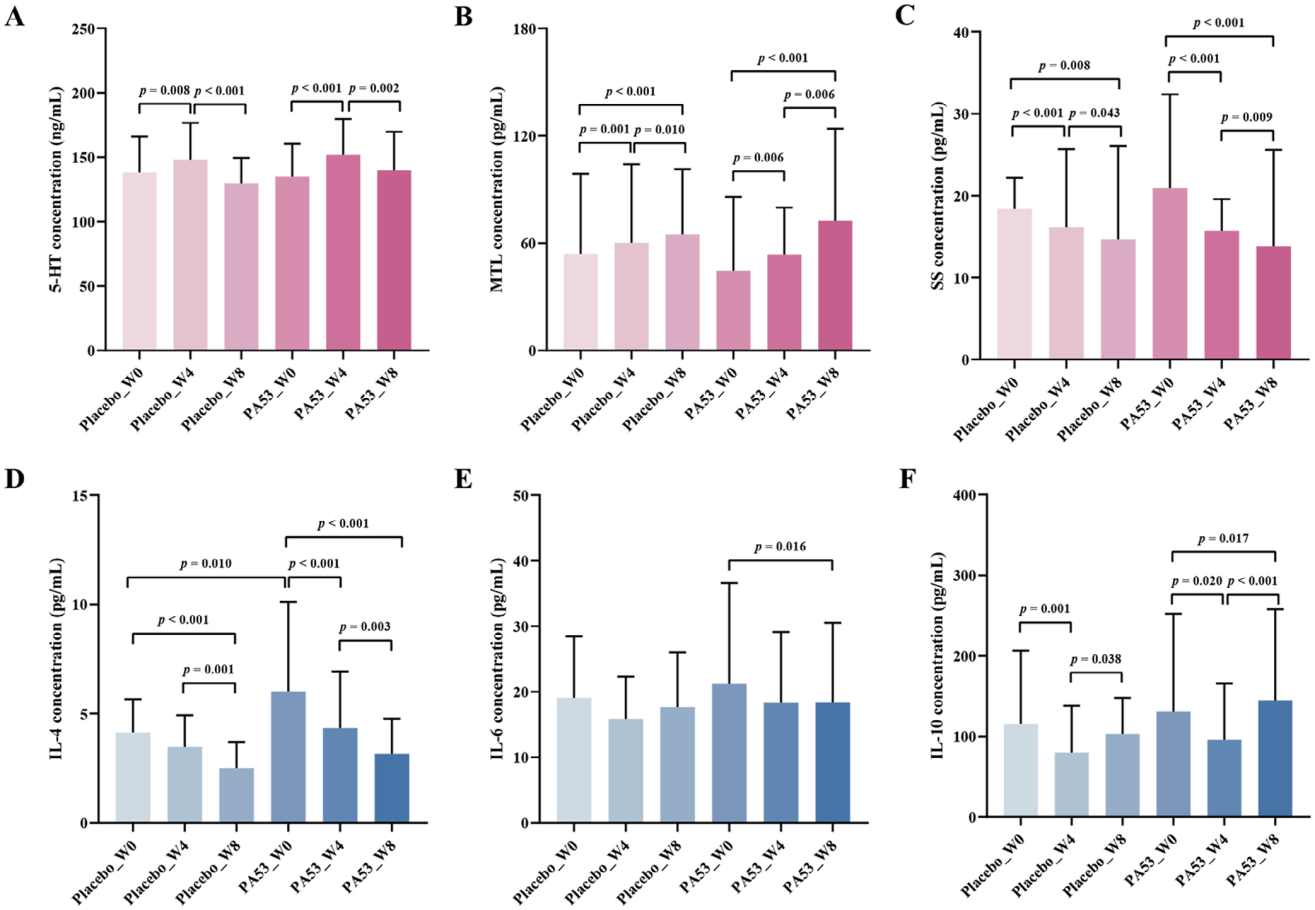
Levels of 5‐HT, MTL, SS, and cytokines (IL‐4, IL‐6, IL‐10) in placebo and PA53 groups. 5‐HT: 5‐Hydroxytryptamine, MTL: Motilin, SS: Somatostatin, IL‐4: Interleukin‐4, IL‐6: Interleukin‐6, IL‐10: Interleukin‐10. Placebo_W0, Placebo_W4, Placebo_W8 represent the time points of Week 0, Week 4, and Week 8 in the placebo group, respectively. PA53_W0, PA53_W4, PA53_W8 represent the time points of Week 0, Week 4, and Week 8 in the PA53 group, respectively. All reported *p*‐values were corrected for multiple comparisons using the Benjamini–Hochberg false discovery rate (FDR) method.

For inflammatory markers, baseline IL‐4 levels were unexpectedly higher in the PA53 group (6.01 ± 4.10 pg/mL vs. 4.12 ± 1.54 pg/mL in placebo, *p* = 0.010). After 8 weeks, IL‐4 levels decreased significantly in both groups to a similar extent (Figure 2D). After adjusting for baseline values using ANCOVA, no significant between‐group treatment effect was observed. Notably, a significant within‐group decrease in IL‐6 was observed in the PA53 group (from 21.19 ± 15.39 pg/mL to 18.30 ± 10.83 pg/mL, *p* = 0.016), but not in the placebo group (from 19.06 ± 9.37 pg/mL to 17.67 ± 8.26 pg/mL, *p* > 0.05). However, the between‐group difference was not significant (*p* > 0.05) (Figure 2E). Regarding IL‐10, time‐dependent changes were observed in both groups during the intervention period. In the PA53 group, IL‐10 levels increased significantly from baseline to week 8 (from 130.82 ± 121.42 pg/mL to 145.02 ± 112.88 pg/mL, *p* = 0.017). In contrast, the placebo group showed no significant change compared with baseline (Figure 2F). No statistically significant between‐group difference was detected at any time point. These results suggest temporal within‐group fluctuations without consistent between‐group treatment effects.

### 3.4 Parameters of the Safety Laboratory

Routine laboratory safety parameters, including albumin, alanine aminotransferase, aspartate aminotransferase, creatinine, uric acid, blood urea nitrogen, and glucose, remained within normal ranges and showed no significant changes from baseline in either group over the 8‐week study period. No significant differences were observed between the PA53 and placebo groups for any of these parameters (all *p* > 0.05, Table 3). Overall, PA53 was well tolerated, with no evidence of adverse effects on standard laboratory safety measures.

**TABLE 3:** Changes in clinical blood measurements.

Variable	Placebo group			PA53 group			Intergroup *p* value		
	W0	W4	W8	W0	W4	W8	W0	W4	W8
Alb	45.41 ± 2.57	45.39 ± 2.9	44.69 ± 2.86	44.5 ± 2.55	44.78 ± 2.79	43.8 ± 3.08	0.114	0.343	0.183
ALT	20.74 ± 9.69	19.56 ± 7.58	20.05 ± 12.79	22.07 ± 9.24	20.55 ± 8.18	20.1 ± 10.86	0.334	0.616	0.922
AST	20.56 ± 5.73	22.08 ± 5.08	21.43 ± 7.19	21.67 ± 4.37	21.69 ± 3.97	22.36 ± 5.86	0.154	0.856	0.233
Crea	64.65 ± 13.17	63.57 ± 13.74	64.11 ± 12.22	63.72 ± 11.96	59.63 ± 9.87	60.65 ± 8.78	0.741	0.352	0.257
UA	275.92 ± 80.48	277.95 ± 89.98	279.18 ± 75.91	287.26 ± 72.8	280.81 ± 54.09	281.71 ± 56.08	0.359	0.283	0.653
Urea	5.21 ± 1.66	4.57 ± 1.4	5.12 ± 1.75	5.22 ± 1.47	5.04 ± 1.31	4.84 ± 1.29	0.982	0.071	0.419
GLU	4.54 ± 0.58	4.72 ± 0.99	5.06 ± 0.84	4.61 ± 0.74	4.81 ± 0.85	5.05 ± 0.94	0.441	0.328	0.688

*Note:* Values are presented as mean ± SD for continuous variables, *p*‐values represent comparisons between the placebo group and the PA53 group at 0, 4, and 8 weeks. *p*‐values were adjusted using the Benjamini–Hochberg false discovery rate (FDR) correction for multiple comparisons. Adjusted *p*‐values < 0.05 were considered statistically significant.

Abbreviations: Alb, Albumin; ALT, alanine aminotransferase; AST, aspartate aminotransferase; GLU, Glucose; UA, uric acid.

### 3.5 Gut Microbiota Diversity and Composition

On stool samples collected at baseline (Week 0), Week 4, and Week 8, we performed 16S rRNA gene sequencing targeting the V3–V4 hypervariable regions. At baseline, the two groups exhibited comparable gut microbial composition, as assessed by both alpha diversity (ACE: *p* = 1.000, FDR‐adjusted *p* = 1.000; Chao: *p* = 0.966, FDR‐adjusted *p* = 1.000; Shannon: *p* = 0.326, FDR‐adjusted *p* = 0.815; Figure 3A–C, Table S1) and beta diversity (PCoA/Adonis: *p* = 0.342; Figure 3D). Community structure at the phylum level was dominated by Bacillota, Bacteroidota, Actinomycetota, and Pseudomonadota (> 99% combined), with no significant differences between groups at any time point (Figures 3E, 4E, 5E). LEfSe analysis at baseline revealed that *Escherichia/Shigella* were relatively enriched in the placebo group and *Sutterella* and *Veillonella* in the PA53 group (Figure 3F, Table S2).

**FIGURE 3:**
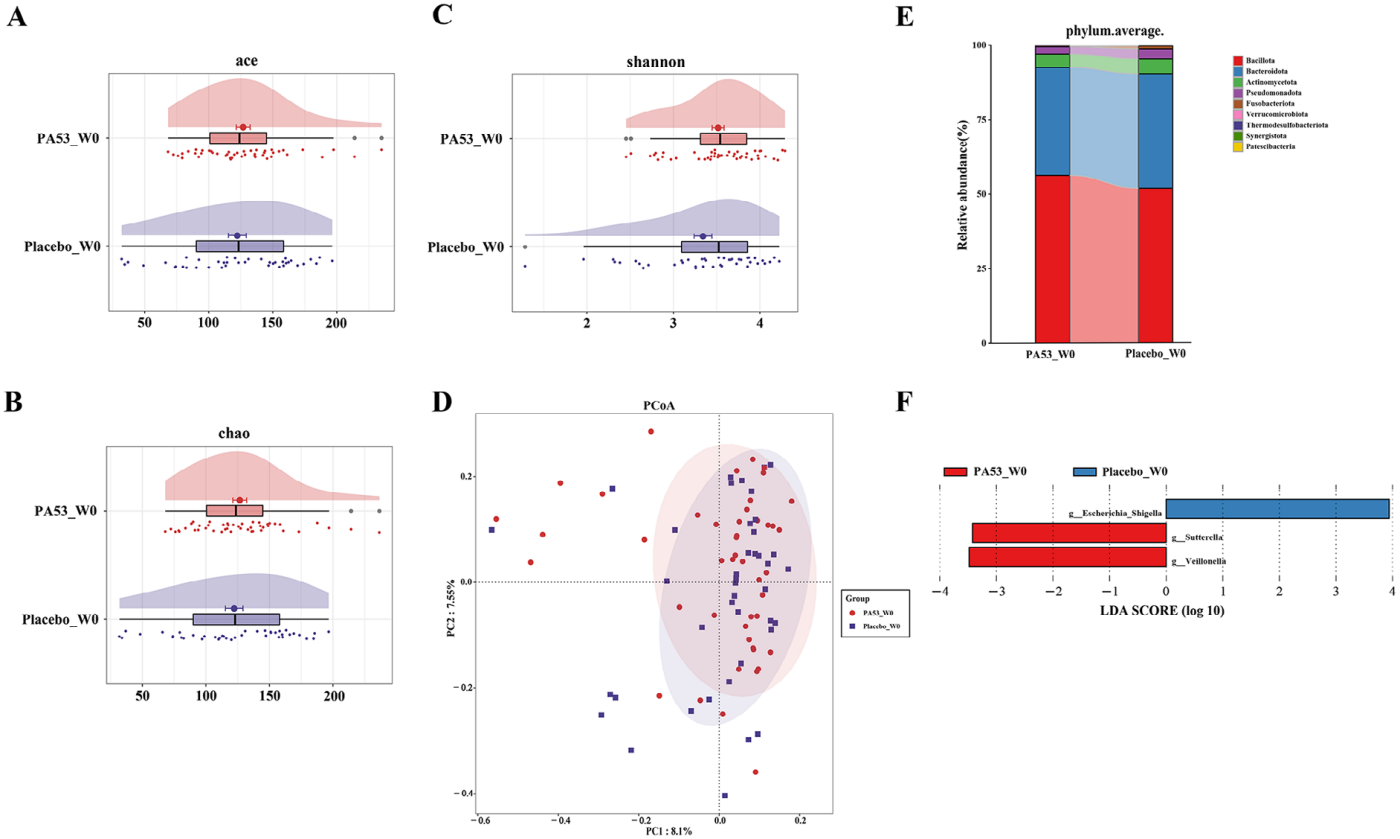
Gut microbiota diversity and composition in the PA53 and placebo groups at baseline. (A–C) Alpha diversity indices (ACE, Chao1, and Shannon). (D) Principal coordinate analysis (PCoA) plot based on Bray‐Curtis's dissimilarity. (E) Gut microbial composition at the phylum level. (F) Histsogram of LDA scores from Linear Discriminant Analysis (LDA) effect size (LEfSe) analysis, highlighting genera with differential abundance between groups. *p*‐values were adjusted using the Benjamini–Hochberg false discovery rate (FDR) correction for multiple comparisons. Differential taxa identified by LEfSe were screened based on an LDA score threshold of > 2.5.

**FIGURE 4:**
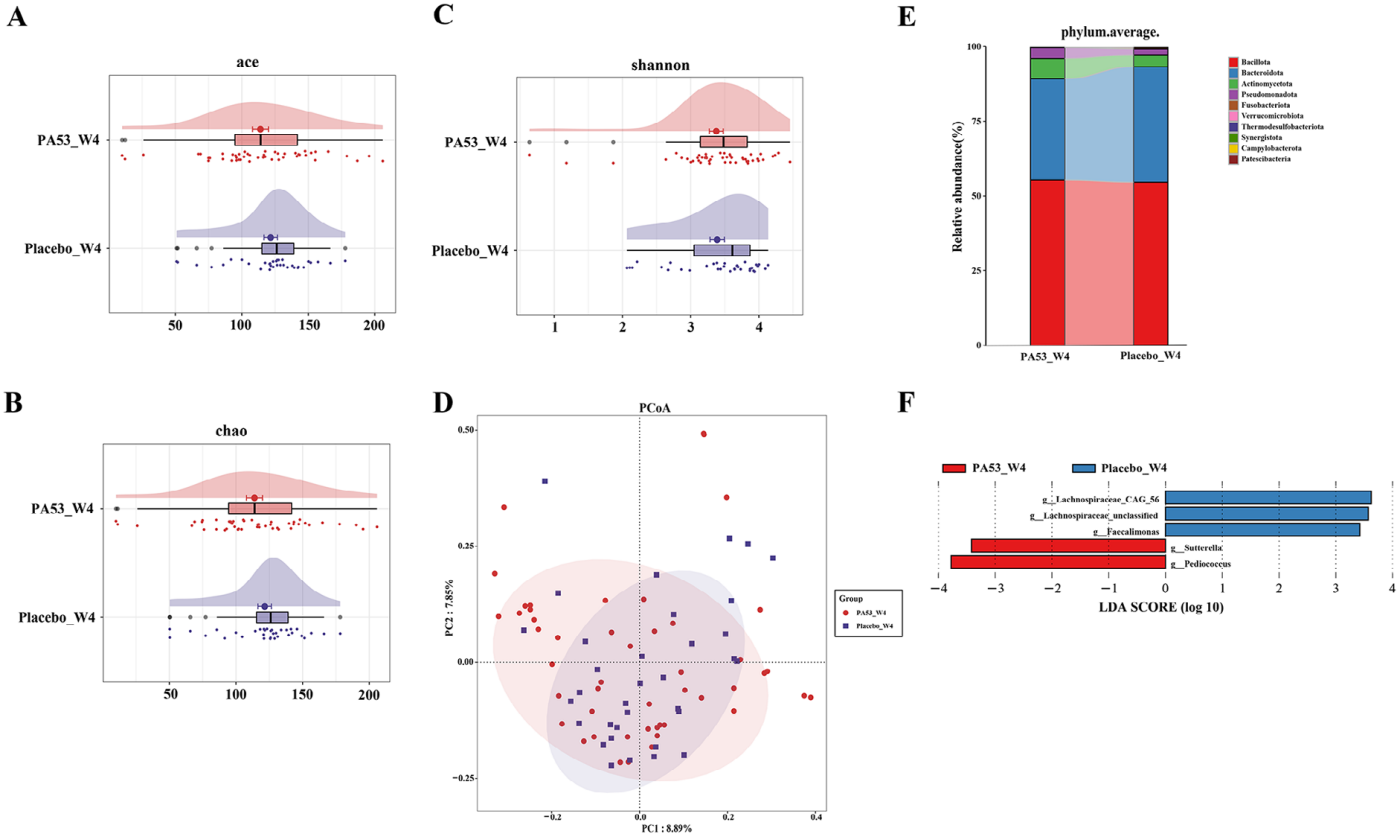
Gut microbiota diversity and composition in the PA53 and placebo groups after 4 weeks of intervention. (A–C) Alpha diversity indices (ACE, Chao1, and Shannon). (D) Principal coordinate analysis (PCoA) plot based on Bray‐Curtis's dissimilarity. (E) Gut microbial composition at the phylum level. (F) Histogram of LDA scores from Linear Discriminant Analysis (LDA) effect size (LEfSe) analysis, highlighting genera with differential abundance between groups. *p*‐values were adjusted using the Benjamini–Hochberg false discovery rate (FDR) correction for multiple comparisons. Differential taxa identified by LEfSe were screened based on an LDA score threshold of > 2.5.

**FIGURE 5:**
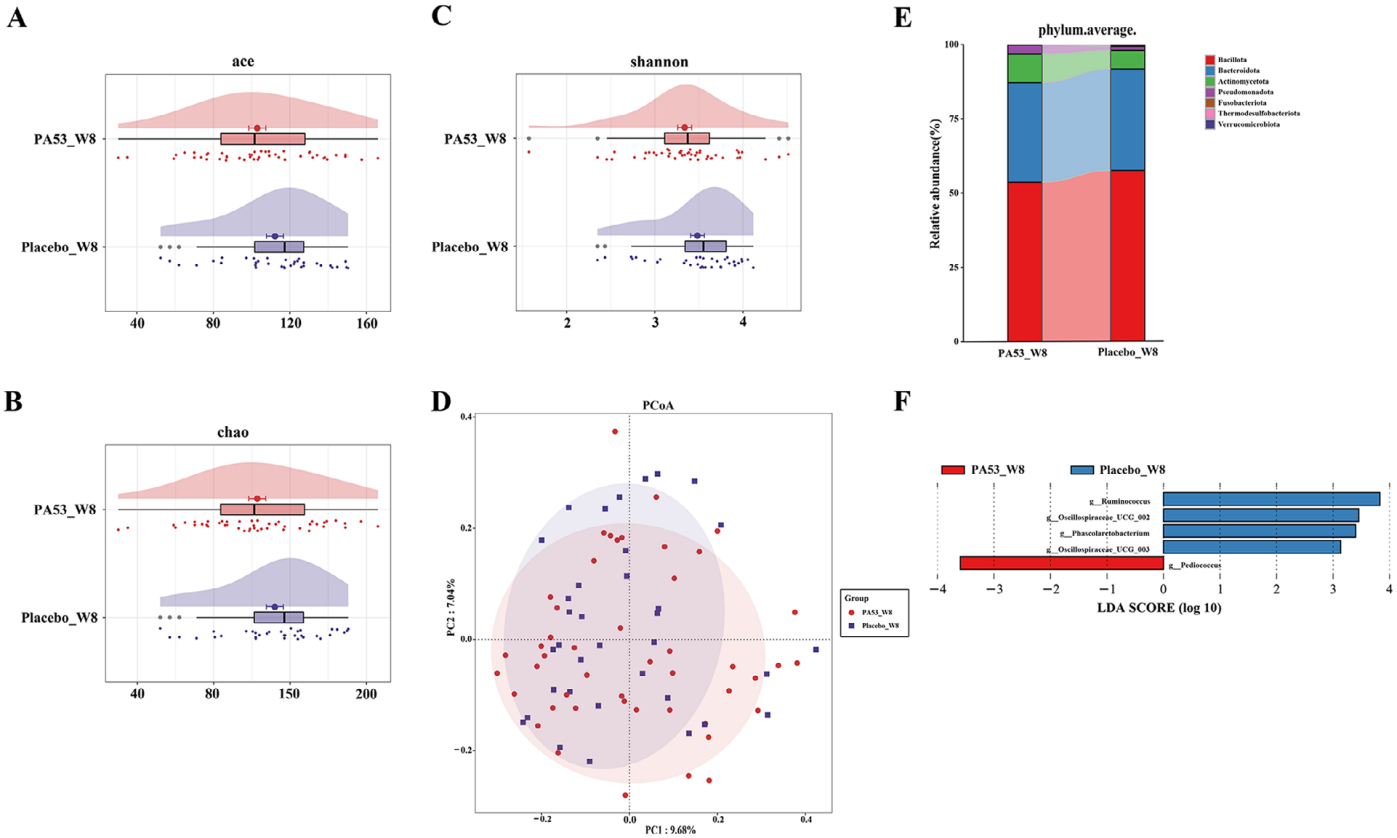
Gut microbiota diversity and composition in the PA53 and placebo groups after 8 weeks of intervention. (A–C) Alpha diversity indices (ACE, Chao1, and Shannon). (D) Principal coordinate analysis (PCoA) plot based on Bray‐Curtis's dissimilarity. (E) Gut microbial composition at the phylum level. (F) Histogram of LDA scores from linear discriminant analysis (LDA) effect size (LEfSe) analysis, highlighting genera with differential abundance between groups. Values were adjusted using the Benjamini–Hochberg false discovery rate (FDR) correction for multiple comparisons. Differential taxa identified by LEfSe were screened based on an LDA score threshold of > 2.5.

After 4 weeks of intervention, overall alpha diversity remained similar between groups (ACE, *p* = 0.253, FDR‐adjusted *p* = 0.421; Chao, *p* = 0.241, FDR‐adjusted *p* = 0.421; Shannon, *p* = 0.760, FDR‐adjusted *p* = 0.950) (Figure 4A–C, Table S1), and beta diversity showed a nonsignificant trend toward separation (adonis, *p* = 0.087) (Figure 4D). Of note, LEfSe indicated a significant enrichment of *Pediococcus* in the PA53 group at week 4, consistent with colonization or increased detection of the administered strain (Figure 4F). Conversely, taxa such as members of the *Lachnospiraceae* and *Faecalimonas* were relatively enriched in the placebo group at this time point (Table S3).

At week 8 (end of intervention), alpha diversity indices again did not differ significantly between PA53 and placebo (ACE, *p* = 0.117, FDR‐adjusted *p* = 0.157; Chao, *p* = 0.117, FDR‐adjusted *p* = 0.157; Shannon, *p* = 0.076, FDR‐adjusted *p* = 0.157) (Figure 5A–C), and differences in beta diversity remained below conventional significance thresholds, although there was an increasing trend toward group separation (adonis, *p* = 0.064) (Figure 5D). LEfSe analysis showed a persistent enrichment of *Pediococcus* in the PA53 group at week 8, while genera such as *Ruminococcus*, *Phascolarctobacterium*, and several *Oscillospiraceae* taxa were more abundant in the placebo group (Figure 5F, Table S4).

### 3.6 Longitudinal Within‐Group Microbiota Dynamics

Longitudinal analysis revealed distinct temporal dynamics between the intervention and placebo groups. In the PA53 group, alpha diversity significantly decreased from baseline to week 8 (ACE, *p* = 0.003, FDR‐adjusted *p* = 0.006; Chao, *p* = 0.003, FDR‐adjusted *p* = 0.006; Shannon, *p* = 0.049, FDR‐adjusted *p* = 0.061) (Figure 6A–C, Table S1). While PCoA plotting indicated visual clustering of samples over time, this structural shift was not statistically significant (adonis, *p* = 0.819) (Figure 6D). Notably, phylum‐level analysis showed a significant time‐dependent increase in Actinomycetota and a decrease in Synergistota in the PA53 group (Figure 6E). LEfSe analysis further identified *Pediococcus* as significantly enriched at week 8 (*p* < 0.001, FDR‐adjusted *p* < 0.001), while *Faecalibacterium* was more abundant at baseline (Figure 6F). In contrast, the placebo group exhibited no significant changes in alpha diversity (ACE, *p* = 0.224, FDR‐adjusted *p* = 0.413; Chao, *p* = 0.214, FDR‐adjusted *p* = 0.413; Shannon, *p* = 0.761, FDR‐adjusted *p* = 0.761) (Figure 7A–C, Table S1), beta diversity (Adonis, *p* = 0.999) (Figure 7D), or phylum‐level composition over the study period (Figure 7E). Collectively, these findings indicate that PA53 administration resulted in a measurable change in microbial community composition and richness over the 8‐week period, although the differences between groups in overall diversity did not reach the usual significance thresholds (Tables S5 and S6).

**FIGURE 6:**
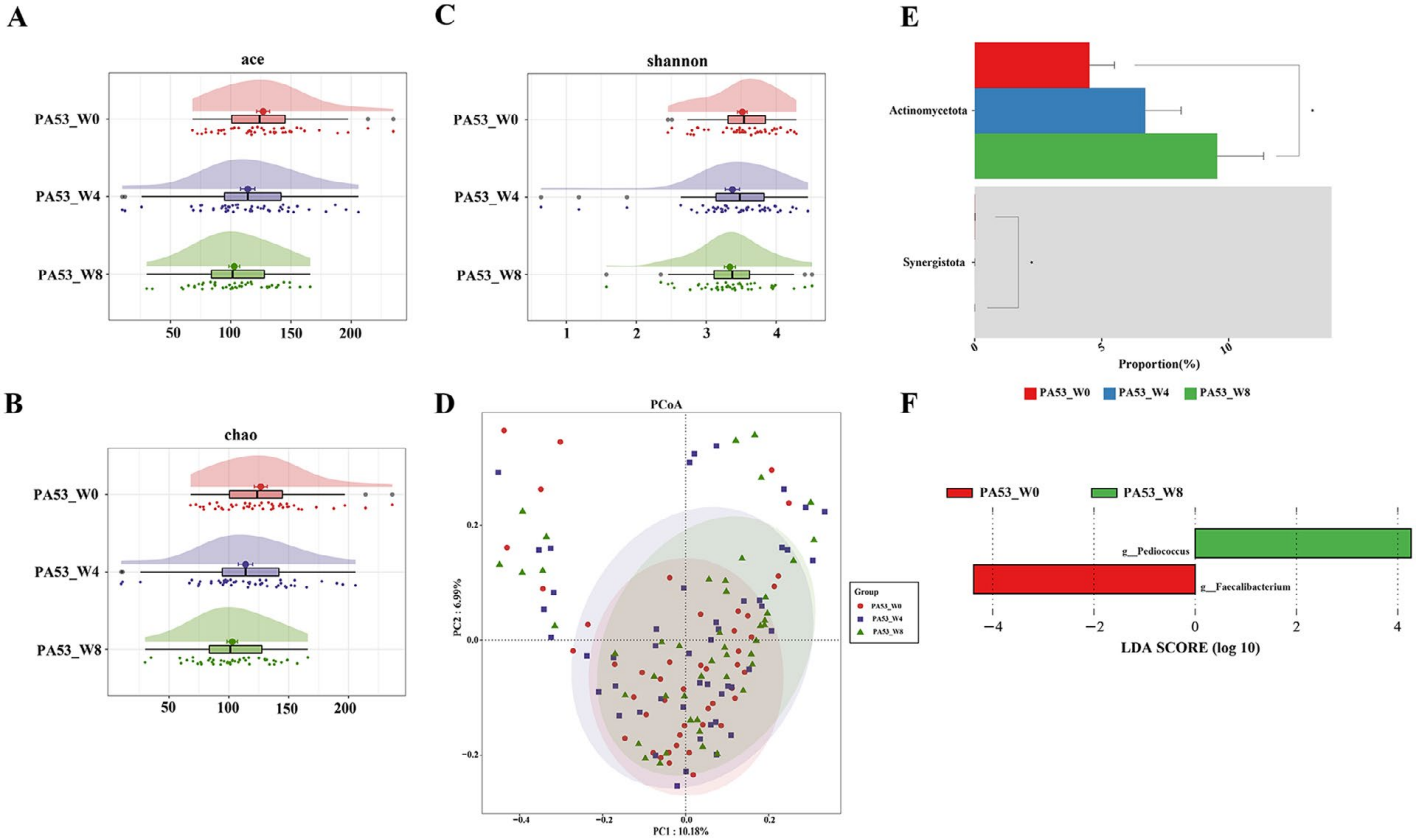
Gut microbiota dynamics in the PA53 group at Weeks 0, 4, and 8 of intervention. (A–C) Alpha diversity indices (ACE, Chao1, and Shannon). (D) Principal coordinates analysis (PCoA) of beta diversity based on Bray‐Curtis's dissimilarity. (E) Relative taxonomic abundance at the phylum level. (F) Differential abundant taxa identified by LEfSe analysis. *p*‐values were adjusted using the Benjamini–Hochberg false discovery rate (FDR) correction for multiple comparisons. Differential taxa identified by LEfSe were screened based on an LDA score threshold of > 2.5.

**FIGURE 7:**
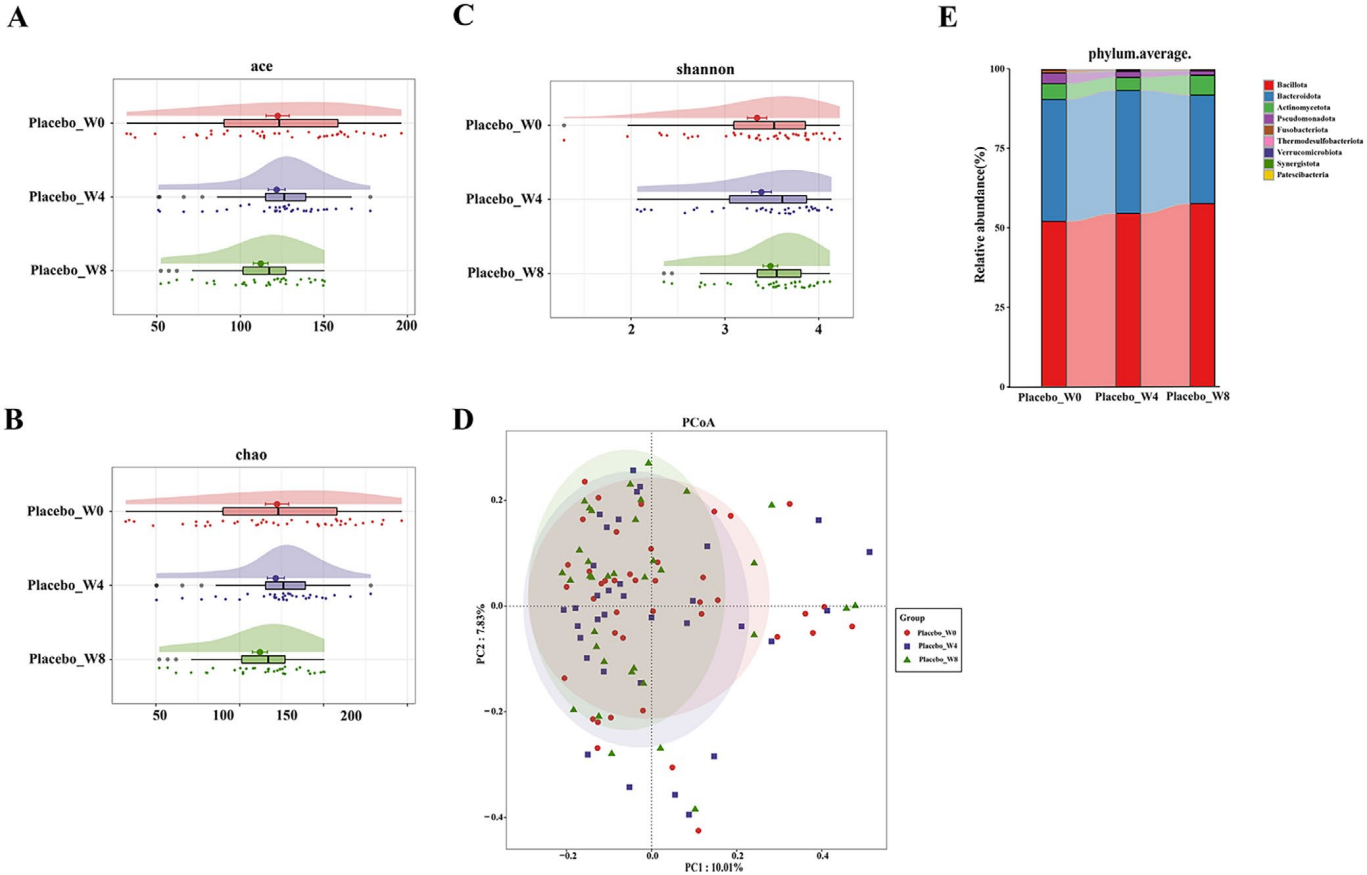
Gut microbiota dynamics in the placebo group at weeks 0, 4, and 8 of intervention. (A)–(C) Alpha diversity indices (ACE, Chao1, and Shannon). (D) Principal coordinates analysis (PCoA) of beta diversity based on Bray‐Curtis dissimilarity. (E) Relative taxonomic abundance at the phylum level.

## 4 Discussion

In this randomized controlled trial, we investigated the effects of 8‐week PA53 supplementation in adults with FC, focusing on changes in constipation symptoms, gut neurotransmitters/hormones, inflammatory markers, and the gut microbiota. PA53 supplementation significantly improved stool form, as evidenced by increased BSFS scores both within the intervention group and compared with placebo. Although the magnitude of change was modest, this directional shift is generally considered clinically meaningful in functional constipation, as stool form is closely associated with patient‐reported comfort, ease of defecation, and perceived bowel regularity. In contrast, bowel movement frequency improved within the PA53 group, but no statistically significant between‐group difference was observed. In addition, PA53 supplementation reduced the level of the pro‐inflammatory cytokine IL‐6, elevated the level of the anti‐inflammatory cytokine IL‐10, and markedly increased the relative abundance of the genus *Pediococcus*. No severe adverse events were observed. These findings indicate that PA53 is both effective and well‐tolerated.

Stool form assessed by BSFS was selected as the primary endpoint due to its higher clinical sensitivity and reproducibility in evaluating constipation severity, whereas SBM was considered a supportive secondary outcome reflecting bowel habit patterns. The improvement in stool consistency is consistent with previous findings suggesting that probiotics may improve stool form in functional constipation, although effects are strain‐specific (Dimidi et al. 2014). In contrast, bowel movement frequency appeared less responsive in the present study, which aligns with the lack of a significant between‐group difference. This suggests that stool form may be more sensitive than bowel movement frequency to short‐term probiotic intervention. Similar dissociations between stool consistency and frequency have been reported previously (Suares and Ford 2011).

In the gut, 5‐HT is primarily synthesized by enterochromaffin cells. It promotes intestinal peristalsis and accelerates content transit by interacting with specific receptors (e.g., 5‐HT_4_ receptors), thereby stimulating enteric neurons and smooth muscle (Yang and Gouaux 2021; Ma et al. 2025). MTL enhances gastrointestinal motility by promoting the formation of motor complexes. Constipation is associated with reduced MTL release, which contributes to decreased gastrointestinal motility (Setchell et al. 2002; Zhang et al. 2023). SS is an inhibitory neurotransmitter that suppresses the release of substance P, relaxes gastrointestinal smooth muscle, reduces peristalsis, and prolongs gut transit time (Wang et al. 2021; Shi et al. 2025). Furthermore, constipation is accompanied by intestinal inflammation, and impaired mucosal barrier function coupled with inflammatory responses are closely linked to the development of constipation symptoms (Hajji et al. 2020; D'Antongiovanni et al. 2020; Cai et al. 2023). Previous studies suggest that probiotic interventions can effectively modulate neurotransmitters, gut hormones, and inflammatory factors in constipated patients (Fan et al. 2025). In this study, both groups exhibited temporal changes in gut hormones, including increases in 5‐HT and MTL and decreases in SS over time, with no significant between‐group differences. These patterns suggest time‐dependent physiological fluctuations during the intervention period rather than definitive treatment‐related neuroendocrine modulation. Compared with the placebo group, the probiotic group showed numerically greater changes in some hormone levels; however, these differences did not reach statistical significance and therefore should be interpreted cautiously. Accordingly, the potential effect of PA53 on intestinal motility via neuroendocrine signaling remains speculative and should be considered hypothesis‐generating rather than confirmed mechanistic evidence. The probiotic group had higher baseline IL‐4 levels, which decreased following the intervention. By week 8, IL‐6 levels decreased significantly in the probiotic group. IL‐10 levels decreased initially and then increased in both groups, with the probiotic group eventually showing levels significantly higher than baseline. These biomarker changes were observed primarily as within‐group temporal patterns, and no consistent between‐group differences were detected across inflammatory markers. Therefore, these findings should be interpreted as exploratory signals rather than confirmed treatment effects.

The pronounced enrichment of *Pediococcus* after supplementation with PA53 indicates successful colonization or at least transient persistence, a prerequisite for a sustained probiotic effect. Animal studies suggest that *P. acidilactici* can increase the abundance of beneficial genera such as *Bifidobacterium* and *Lactobacillus*, while potentially pathogenic Enterobacteriaceae decrease (Li et al. 2023; Qiao et al. 2021). Such microbial shifts can increase the production of SCFAs, lower luminal pH, and stimulate peristalsis, thereby improving symptoms of constipation (Qiao et al. 2021; Tian et al. 2024). PA53 was well tolerated, and no serious adverse events were reported. This result is consistent with the safety profiles of other *P. acidilactici* strains, such as J9, which showed no toxicity in animal studies and even reduced *Helicobacter pylori* adhesion (Lee et al. 2020). The absence of clinically significant alterations in biochemical markers further supports the suitability of PA53 as a dietary supplement for individuals with functional constipation.

In summary, the findings of this study, together with existing evidence, suggest that PA53 may be associated with potential mechanisms or pathways underlying its effects on constipation, including: (1) a possible role in modulating gut microbial composition and promoting beneficial taxa enrichment; (2) potential involvement in intestinal motility regulation, possibly through gut–brain axis‐related neuroendocrine signaling; and (3) a potential contribution to the modulation of intestinal inflammatory responses. However, given the lack of consistent between‐group differences in inflammatory markers and other biomarkers, these interpretations remain exploratory and hypothesis‐generating rather than confirmatory evidence of causal mechanisms, and require further validation in larger, well‐powered clinical trials.

This study has several limitations. First, the relatively small sample size may have reduced the statistical power to detect between‐group differences in outcomes such as BSFS and SBM. In particular, the use of categorical SBM data may have further reduced statistical sensitivity compared with continuous measures. Second, the 8‐week intervention period might be insufficient to observe long‐term microbial colonization restructuring or the sustainability of symptom improvement. Furthermore, the lack of strict control over dietary intake may introduce potential confounding factors. In addition, dextrin, used as placebo in this study, is a soluble dietary fiber that may exert mild effects on bowel function; therefore, a completely inert placebo cannot be guaranteed, which may have led to an underestimation of the observed treatment effects. On a mechanistic level, key indicators such as SCFA levels, tight junction protein expression, and mucosal immune markers were not assessed. Future studies should involve larger‐scale, multi‐center randomized controlled trials with longer follow‐up periods and more comprehensive mechanistic endpoints to further validate and extend these findings.

## 5 Conclusion

The findings indicate that 8‐week supplementation with *P*. *acidilactici* PA53 significantly improves stool form and is associated with beneficial modulation of gut microbiota and inflammatory markers in adults with functional constipation. However, bowel movement frequency showed improvement only within the intervention group, without a statistically significant between‐group difference. These results suggest that PA53 may have a more pronounced effect on stool consistency than on evacuation frequency and appears to be safe and well tolerated.

## Author Contributions

**Yuhan Xia:** writing – original draft, writing – review and editing, project administration, methodology, conceptualization. **Yongmei Cai:** methodology, conceptualization, writing – review and editing, writing – original draft. **Yongwei Zhang:** conceptualization, methodology, writing – review and editing, writing – original draft. **Rui Cai:** methodology, conceptualization, writing – original draft. **Hanshu Liu:** formal analysis, writing – review and editing. **Zhiwen Qiang:** formal analysis, writing – review and editing.

## Funding

The authors have nothing to report.

## Disclosure

Wecare Probiotics Co. Ltd. provided the investigational probiotic product (PA53) and placebo used in this study.

The authors did not use generative artificial intelligence (AI) tools in the preparation of this manuscript.

## Conflicts of Interest

The authors declare no conflicts of interest.

## Supporting information


**Table S1:** Alpha diversity analysis of gut microbiota in the PA53 and placebo groups.
**Table S2:** Differential abundance analysis of gut microbiota at the genus level between the PA53 and placebo groups at baseline.
**Table S3:** Differential abundance analysis of gut microbiota at the genus level between the PA53 and placebo groups after 4 weeks of intervention.
**Table S4:** Differential abundance analysis of gut microbiota at the genus level between the PA53 and placebo groups after 8 weeks of intervention.
**Table S5:** Differential abundance analysis of gut microbiota at the genus level within the PA53 group from baseline to weeks 4 and 8.
**Table S6:** Differential abundance analysis of gut microbiota at the genus level within the placebo group from baseline to weeks 4 and 8.

## Data Availability

The data that support the findings of this study are available from the corresponding author upon reasonable request.
